# Correction to *Lancet HIV* 2019; 6: e105–15

**DOI:** 10.1016/S2352-3018(20)30029-1

**Published:** 2020-01-28

*The Collaborative Initiative for Paediatric HIV Education and Research (CIPHER) Global Cohort Collaboration. Incidence of switching to second-line antiretroviral therapy and associated factors in children with HIV: an international cohort collaboration.* Lancet HIV *2019; **6**: e105–15*—The Summary Funding line has been revised to include UK Medical Research Council and this Article has been made Open Access. These corrections were made on Jan 28, 2020.

